# Multimodal Pharmacologic Strategies for Chronic Cancer Pain: A Systematic Review

**DOI:** 10.7759/cureus.114909

**Published:** 2026-08-21

**Authors:** Savannah Q Thirza, Amanda Squire, Richard Berman, Ashique Ahamed

**Affiliations:** 1 Supportive Care, The Christie NHS Foundation Trust, Manchester, GBR; 2 Division of Cancer Sciences, Manchester Academic Health Science Centre, University of Manchester, Manchester, GBR

**Keywords:** adjuvant analgesics, cancer pain, multimodal analgesia, opioids, systematic review

## Abstract

Cancer pain remains inadequately controlled in some patients despite opioid therapy, particularly when neuropathic or mixed pain mechanisms are present. This systematic review evaluated the efficacy and safety of non-opioid or adjuvant analgesics used alongside opioids in adults with chronic cancer pain. MEDLINE, Embase, and Cochrane Central Register of Controlled Trials (CENTRAL) were searched for English-language randomized controlled trials published from January 2010 to August 2025. Two reviewers independently selected studies, extracted data, and assessed risk of bias using Cochrane Risk of Bias 2 (RoB 2). Ten trials involving 1,344 participants were included. In one trial, duloxetine was associated with clinically meaningful pain-response rates, although its primary mean pain-score analysis was not statistically significant. Low-dose gabapentin plus imipramine reduced pain and rescue-morphine use. Pregabalin reduced the morphine dose required to maintain pain control in one crossover trial, while other pregabalin studies showed numerical or non-significant benefits. Neither nabiximols trial met its primary efficacy endpoint, and nefopam did not significantly improve pain response or morphine consumption. Risk of bias was low in two trials, raised some concerns in one, and was high in seven. Overall, the evidence remains heterogeneous and insufficient to support a standard multimodal pharmacological regimen. Selected antidepressant and gabapentinoid strategies, particularly in neuropathic cancer pain, may benefit selected patients.

## Introduction and background

Cancer-related pain remains a major clinical challenge across the cancer trajectory, affecting approximately 55% of patients receiving anticancer treatment and 66% of those with advanced, metastatic, or terminal disease, while around 39% experience pain following curative treatment [1]. Pain can occur from diagnosis and active treatment through to survivorship and may adversely affect physical function, emotional well-being, and overall quality of life [1,2]. Current European Society for Medical Oncology (ESMO) and National Comprehensive Cancer Network (NCCN) guidelines therefore recommend comprehensive, individualized assessment and multimodal management of cancer-related pain, incorporating opioid, non-opioid or adjuvant, and non-pharmacological approaches where appropriate [2,3]. Almost 3.5 million people are currently estimated to be living with cancer in the United Kingdom, further increasing the importance of effective long-term pain management [4].

Cancer-related pain may arise directly from tumor involvement or as a consequence of surgery, radiotherapy, or systemic anticancer treatment [2,5]. Its underlying mechanisms may be nociceptive, neuropathic, or mixed, with nociceptive pain encompassing somatic and visceral sources [5]. Neuropathic pain may result from tumour-related nerve compression or treatment-induced nerve injury and is commonly described as burning, shooting, or electric-shock-like pain. Nociceptive pain may originate from bone, soft tissue, or visceral structures and is more commonly described as aching, pressure-like, or throbbing [5]. These mechanisms frequently overlap, supporting an individualized, mechanism-based approach to management [5].

The World Health Organization analgesic ladder remains an established framework for cancer-pain management, with opioids central to the treatment of moderate-to-severe pain [6,7]. However, some patients continue to experience persistent or breakthrough pain despite opioid therapy, while dose escalation may be limited by adverse effects such as constipation, nausea, sedation, cognitive impairment, tolerance, and physical dependence [5,7]. Opioid therapy alone may therefore not provide an optimal balance between analgesia and tolerability for every patient.

These limitations have increased interest in multimodal pharmacological strategies combining opioids with non-opioid or adjuvant analgesics targeting different pain mechanisms [8,9]. Antidepressants and gabapentinoids are commonly used for neuropathic cancer pain by targeting pain pathways not primarily addressed by opioids, while cannabinoids and other centrally acting agents have also been evaluated as adjunctive therapies [8-10]. Combination pharmacotherapy may improve analgesia, reduce opioid requirements, or permit lower doses of individual drugs; however, it may also increase treatment burden and adverse effects [9,10].

Previous systematic reviews have evaluated adjuvant analgesics or combinations of non-opioid drugs for cancer pain, but found limited and heterogeneous evidence supporting specific agents or combinations [11,12]. The effectiveness and safety of non-opioid or adjuvant analgesics used specifically alongside opioids for chronic cancer-related pain therefore remain uncertain, particularly regarding their potential to improve analgesia, reduce opioid requirements, and improve functional and patient-centered outcomes [11,12]. This systematic review evaluated the efficacy and safety of multimodal pharmacological strategies combining opioids with non-opioid or adjuvant analgesics, including their effects on pain response, opioid consumption, rescue-analgesic use, physical function, quality of life, sleep, tolerability, and adverse events.

This work was previously presented as an oral rapid e-poster at the Multinational Association of Supportive Care in Cancer (MASCC) Annual Meeting, Melbourne, Australia, July 25, 2026.

## Review

Methodology

This systematic review was conducted in accordance with the Preferred Reporting Items for Systematic Reviews and Meta-Analyses (PRISMA) 2020 statement and was prospectively registered with the International Prospective Register of Systematic Reviews (PROSPERO; CRD420251120781) on August 6, 2025. The review question and eligibility criteria were structured using the Population, Intervention, Comparator, and Outcome framework. The population comprised adults aged 18 years or older with chronic cancer-related pain that was inadequately controlled by, or previously treated with, opioid analgesia. Eligible interventions involved a multimodal pharmacological regimen incorporating at least one non-opioid or adjuvant analgesic alongside opioid therapy. Eligible comparators included opioid monotherapy, opioid plus placebo, or an alternative opioid-non-opioid regimen using different agents or dosing strategies. The primary outcomes were cancer-related pain intensity, pain relief, or clinically meaningful pain response. Secondary outcomes included opioid consumption, rescue-analgesic use, physical function, quality of life, sleep, patient satisfaction, tolerability, and adverse events. 

Eligibility Criteria

Randomized controlled trials were eligible if they included adults aged 18 years or older with chronic cancer-related pain attributable either directly to cancer or to cancer treatment. Chronic cancer-related pain was considered eligible when it was explicitly described as chronic, persistent, or longstanding, or when the clinical context indicated established ongoing pain requiring regular analgesic therapy. This included pain described as opioid-refractory, inadequately controlled despite opioid therapy, or requiring opioid dose escalation. 

Eligible interventions evaluated multimodal pharmacological therapy in which a non-opioid or adjuvant analgesic was administered alongside opioid therapy. Eligible comparators included opioid monotherapy, opioid plus placebo, or an alternative opioid-non-opioid regimen using different agents or dosing strategies.

Studies were required to report cancer-related pain intensity or pain relief using a validated pain measure or a clinically meaningful pain-response threshold. Secondary outcomes of interest included opioid consumption, rescue-analgesic use, physical function, quality of life, sleep, patient satisfaction, tolerability, and adverse events.

Studies evaluating exclusively acute pain, including postoperative pain or transient treatment-related pain, were excluded. Where pain duration was not explicitly reported, chronic cancer pain was defined as established cancer-related pain requiring ongoing regular analgesic therapy; no minimum duration threshold was applied. Studies conducted within supportive or palliative care services were eligible where participants were receiving ongoing cancer treatment or survivorship care; however, studies exclusively involving patients receiving terminal, hospice, end-of-life, or last-days-of-life care were excluded, as pharmacological management in these settings may primarily reflect short-term symptom control and comfort-focused care rather than longer-term management of chronic cancer pain. Pediatric studies, non-randomized studies, uncontrolled studies, studies without an eligible comparator, and studies evaluating exclusively non-pharmacological, interventional, or procedural pain-management strategies were also excluded. Only full-text articles published in English were eligible.

Randomized controlled trials were selected because the review aimed to evaluate comparative evidence regarding the efficacy and safety of multimodal pharmacological treatment. Acute and postoperative pain studies were excluded because these represent clinically distinct pain settings with different treatment goals, durations, and analgesic requirements.

Search Strategy

A systematic literature search was conducted in MEDLINE and Embase via Ovid and in the Cochrane Central Register of Controlled Trials (CENTRAL) via the Cochrane Library. The searches were last conducted on August 20, 2025, and covered records published from January 1, 2010, to August 20, 2025. The 2010 lower date limit was selected to focus the review on contemporary evidence most relevant to current cancer-pain management and clinical practice. The complete database-specific search strategies are provided in Appendices A-C.

The search combined controlled vocabulary, including Medical Subject Headings and database-specific indexing terms, with free-text terms relating to cancer, chronic cancer-related pain, opioids, non-opioid or adjuvant analgesics, combination pharmacotherapy, and multimodal analgesia. Synonymous terms within each concept were combined using the Boolean operator OR, and the separate concepts were combined using AND. The strategy was developed with input from an expert medical librarian (DL) and adapted to the syntax and indexing structure of each database. Unpublished studies and gray literature were not searched, in accordance with the registered protocol. In addition, the search required opioid-related terminology to reflect the prespecified review question, which may have missed eligible studies where background opioid therapy was not clearly reported in the title, abstract, or indexing. The search strategy was not formally validated against a prespecified set of sentinel studies.

Study Selection

All records retrieved through the database searches were imported into Rayyan (Rayyan Systems Inc., Cambridge, MA; web application). Duplicate records were identified and removed before screening.

Two reviewers (ST and AS) independently screened the titles and abstracts of all retrieved records against the prespecified eligibility criteria. Full-text reports were retrieved when a record appeared potentially eligible or when eligibility could not be determined reliably from the title and abstract alone.

At full-text review, studies were included only if they involved adults with established chronic cancer-related pain, used a randomized controlled design, evaluated an eligible opioid-non-opioid or adjuvant pharmacological regimen, included an eligible comparator, and reported an eligible pain outcome. Reports were excluded if they evaluated acute or postoperative pain, used an ineligible intervention or comparator, were not eligible full-text randomized controlled trials, duplicated data from an already represented study population, or otherwise failed to meet the prespecified eligibility criteria. Disagreements at the title-and-abstract or full-text screening stages were resolved through discussion and consensus. Reasons for exclusion were recorded for all reports excluded following full-text assessment.

**Figure 1:**
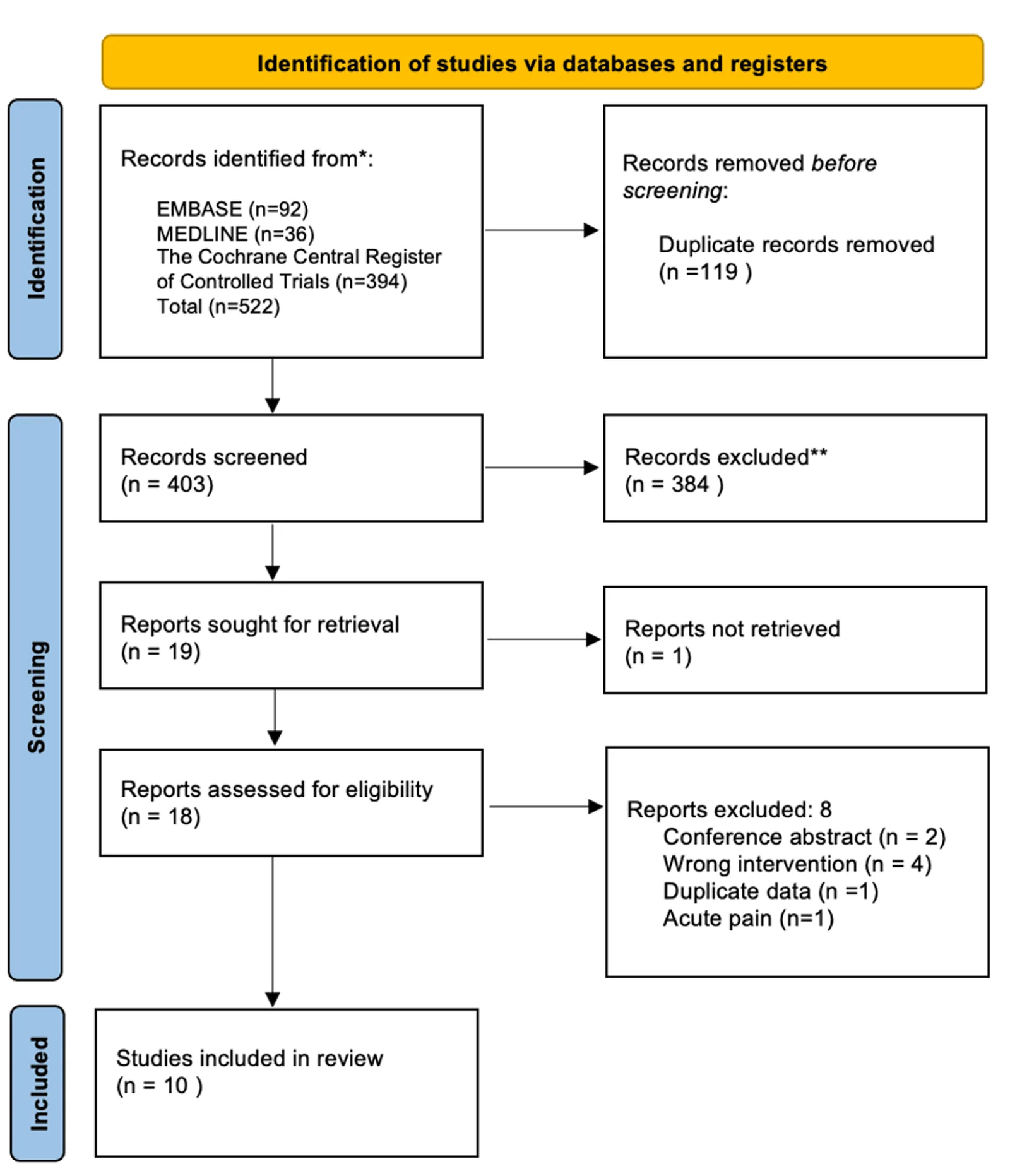
PRISMA 2020 flow diagram showing the identification, screening, eligibility assessment, and inclusion of studies. The figure was created using the official PRISMA 2020 flow diagram template. PRISMA, Preferred Reporting Items for Systematic Reviews and Meta-Analyses

The searches identified 522 records. After removal of 119 duplicates, 403 records underwent title and abstract screening, of which 384 were excluded. Nineteen reports were sought for full-text retrieval; one could not be obtained, leaving 18 reports for eligibility assessment. Eight reports were excluded at full-text review: two were conference abstracts without eligible full-text publications, four evaluated ineligible interventions, one reported duplicate data from an already represented study population, and one evaluated acute rather than chronic cancer-related pain. Ten randomized controlled trials met all eligibility criteria and were included in the review. The study-selection process and reasons for exclusion are presented in a PRISMA 2020 flow diagram (Figure 1).

Data Extraction

Data were independently extracted by two reviewers (ST and AS) using a standardized data extraction form developed in Microsoft Excel for Microsoft 365 (2025 version; Microsoft Corporation, Redmond, WA).

Extracted data included study characteristics, sample size, participant characteristics, cancer type and pain characteristics, intervention and comparator regimens, trial duration, primary and secondary outcomes, and reported adverse events. The primary outcomes were cancer-related pain intensity, pain relief, or clinically meaningful pain response, as defined by the original trial. Secondary outcomes included opioid requirements, rescue-analgesic use, physical function, quality of life, sleep, patient satisfaction, tolerability, and adverse events. Discrepancies in extracted data were resolved through discussion and consensus between the two reviewers.

Risk-of-Bias Assessment

**Figure 2:**
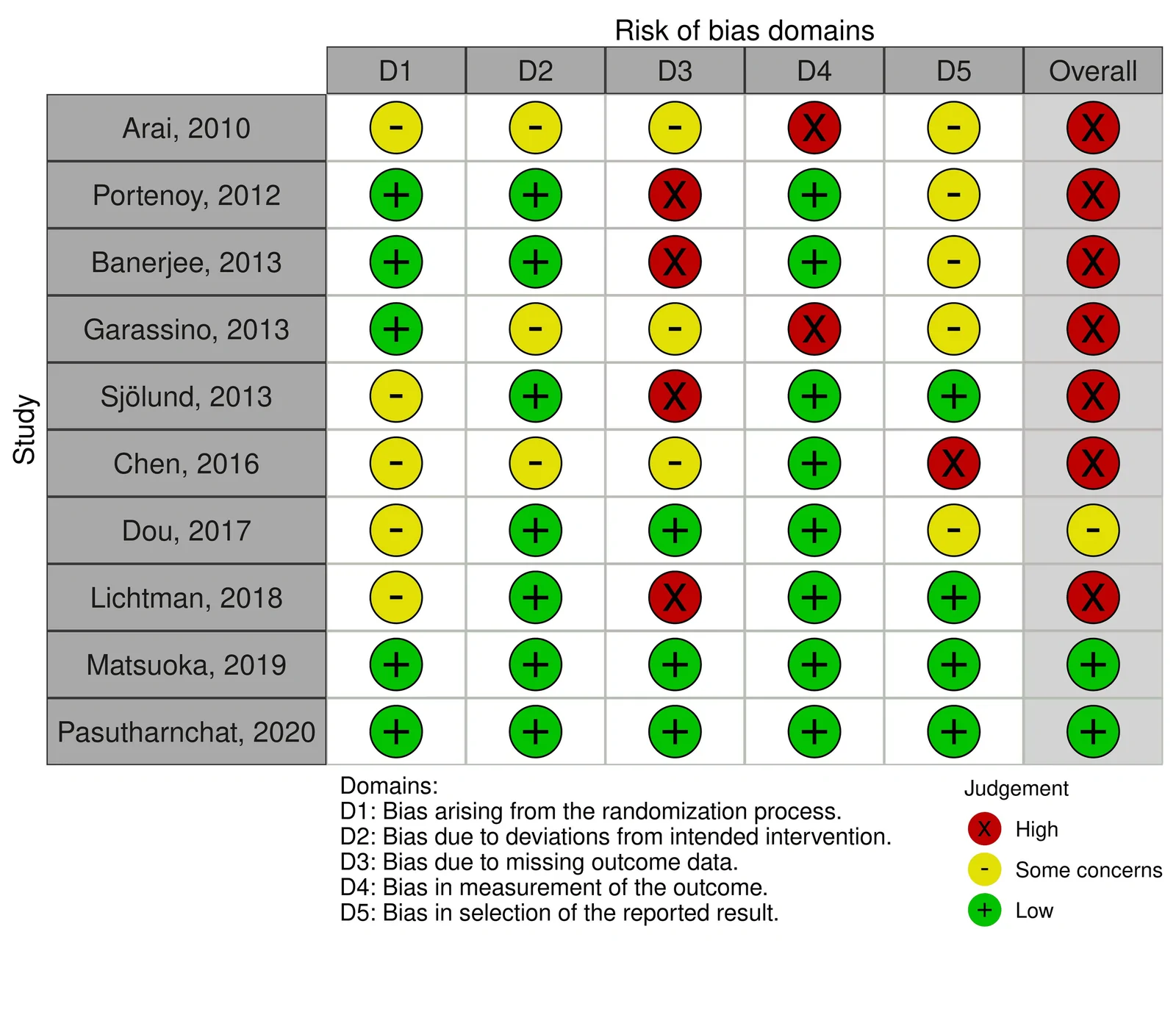
Traffic-light plot showing the Risk of Bias 2 assessment of the 10 randomized controlled trials. The figure was generated using the robvis web application (Risk-of-Bias VISualization) [14].

Two reviewers independently assessed each included trial using the Cochrane RoB 2 tool [13]. Assessments were completed using the Cochrane RoB 2 Excel tool, version 22 August 2019, for individually randomized parallel-group trials, with the corresponding crossover version used where applicable. Reviewers independently completed the signaling questions, recorded supporting information, and reviewed the algorithm-generated domain-level judgments. Overall judgments of low risk of bias, some concerns, or high risk of bias were assigned in accordance with RoB 2 guidance. Disagreements were resolved through discussion and consensus. The final domain-level and overall judgments were entered into the robvis web application (Risk-of-Bias VISualization) to generate the traffic-light plot presented in Figure 2 [14]. Detailed study-level domain judgments and rationale are provided in Appendix D.

Synthesis Methods

A narrative synthesis was undertaken because substantial clinical and methodological heterogeneity across studies precluded meaningful quantitative pooling. Studies differed in pharmacological interventions, comparator regimens, cancer populations, pain mechanisms, baseline opioid treatment, treatment duration, outcome measures, responder definitions, and assessment time points.

Results were grouped according to pharmacological strategy and reported using the effect measures presented in the original studies, including mean or median pain scores, changes from baseline, responder proportions, odds ratios, confidence intervals, and measures of opioid consumption. No meta-analysis was performed because no clinically coherent comparison contained a sufficient number of studies with compatible populations, interventions, comparators, outcomes, and assessment time points. Consequently, statistical measures of heterogeneity, such as *I*², were not calculated.

Results 

The database searches identified 522 records from MEDLINE and Embase via Ovid and CENTRAL via the Cochrane Library. After removal of 119 duplicates, 403 records underwent title and abstract screening, of which 384 were excluded. Nineteen reports were sought for full-text retrieval; one report could not be obtained, leaving 18 reports for eligibility assessment. Eight reports were excluded because they were conference abstracts (*n* = 2), evaluated an ineligible intervention (*n *= 4), reported duplicate data (*n* = 1), or assessed acute pain (*n* = 1). Ten randomized controlled trials met the eligibility criteria and were included in the review. The study selection process is presented in Figure 1.

The 10 included trials randomized between 40 and 397 participants, with treatment periods ranging from 48 hours to six months. The interventions were grouped into four clinical strategies: antidepressant and gabapentin-based combinations; pregabalin-opioid strategies; nabiximols added to opioid therapy; and intravenous nefopam added to morphine. Comparator regimens included placebo alongside opioid therapy, an alternative adjuvant-opioid combination, or a different dose-escalation strategy. Considerable variation was observed in cancer populations, pain mechanisms, background opioid treatment, intervention and comparator regimens, outcome definitions, and assessment time points. Study characteristics are summarized in Table 1, and efficacy and safety outcomes are presented in Table 2. Because the findings were synthesized narratively and no meta-analysis was undertaken, statistical heterogeneity measures such as *I*² were not calculated.

**Table 1:** Characteristics of included studies. ITT, intention-to-treat; IV, intravenous; NSAIDs, non-steroidal anti-inflammatory drugs; NSCLC, non-small cell lung cancer; PCA, patient-controlled analgesia; RCT, randomized controlled trial; VAS, visual analog scale

Author	Study design	Population	Sample size	Intervention	Comparator	Treatment duration
Matsuoka et al. (2019) [15]	Multicenter, double-blind, placebo-controlled, parallel-group RCT	Cancer-related neuropathic pain inadequately responsive to, or intolerant of, opioid-pregabalin therapy	70 randomized; 67 evaluable	Duloxetine 20 mg/day, increased to 40 mg/day, alongside existing pregabalin and opioid therapy	Placebo alongside existing pregabalin and opioid therapy	10 days
Arai et al. (2010) [16]	Four-arm, randomized, open-label trial	Cancer-related neuropathic pain inadequately controlled by opioids and NSAIDs	52 randomized	Low-dose gabapentin plus imipramine	Low-dose gabapentin, higher-dose gabapentin, or imipramine alone; background opioids unchanged	7 days
Banerjee et al. (2013) [17]	Single-center, open-label, parallel-group RCT	Neuropathic pain associated with stage III head and neck, breast, lung, or cervical cancer	88 randomized; 76 completed	Gabapentin plus tramadol	Amitriptyline plus tramadol	6 months
Chen et al. (2016) [18]	Single-center, randomized, double-blind, parallel-group study	Adults with severe cancer pain (VAS > 7) involving colorectal, lung, prostate, gastric, or cervical cancer	70 enrolled; 60 analyzed	Gabapentin, titrated up to 2,700 mg/day, plus controlled-release oxycodone	Placebo plus controlled-release oxycodone	6 months
Sjölund et al. (2013) [19]	International multicenter, double-blind, placebo-controlled, parallel-group RCT	Moderate-to-severe cancer-induced bone pain from confirmed bone metastases despite optimized stable opioid therapy	152 randomized; 118 completed	Flexible-dose pregabalin 100-600 mg/day added to stable opioid therapy	Placebo added to stable opioid therapy	28 days, followed by a 6-day taper
Dou et al. (2017) [20]	Single-center, double-blind, placebo-controlled crossover RCT	Adults with severe neuropathic cancer pain receiving morphine therapy	40 randomized; 38 completed; 40 included in ITT analysis	Pregabalin 150-300 mg/day plus morphine	Placebo plus morphine	Two 2-week periods separated by a 1-week washout
Garassino et al. (2013) [21]	Multicenter, randomized phase II trial	Neuropathic pain associated with NSCLC, breast, colorectal, or other cancers	75 randomized; 67 completed	Fixed-dose oxycodone with escalating pregabalin	Fixed-dose pregabalin with escalating oxycodone	14 days
Portenoy et al. (2012) [22]	Multicenter, double-blind, placebo-controlled, graded-dose RCT	Advanced cancer with chronic moderate-to-severe pain inadequately controlled by stable opioid therapy	360 randomized; 263 completed	Low-, medium-, or high-dose nabiximols added to stable opioid therapy	Placebo added to stable opioid therapy	5 weeks
Lichtman et al. (2018) [23]	Multicenter, double-blind, placebo-controlled, phase III RCT	Advanced cancer with chronic pain inadequately controlled by optimized strong opioid therapy	397 randomized; 291 completed	Self-titrated nabiximols oromucosal spray added to existing opioids	Placebo spray added to existing opioids	5 weeks
Pasutharnchat et al. (2020) [24]	Double-blind, placebo-controlled, parallel-group RCT	Adults with moderate-to-severe cancer pain requiring IV morphine PCA	40 randomized and analyzed	Intravenous nefopam 20 mg every 8 hours for three doses plus morphine PCA	Saline placebo plus morphine PCA	48 hours

**Table 2:** Summary of study outcomes and adverse events. ^a^Chen et al. [18] did not clearly identify a prespecified primary pain endpoint or report detailed comparative VAS results. ^b^Sjölund et al. [19] terminated recruitment early; therefore, outcomes were reported descriptively without formal between-group hypothesis testing. ^c^Dou et al. [20] designated morphine-dose reduction as the primary outcome. CI, confidence interval; ITT, intention-to-treat; NRS, numeric rating scale; OR, odds ratio; PGIC, Patient Global Impression of Change; VAS, visual analog scale; VRS, verbal rating scale

Authors	Primary pain outcome	Opioid or rescue-analgesic outcomes	Secondary outcomes	Adverse events
Matsuoka et al. (2019) [15]	Mean pain score was 4.03 with duloxetine versus 4.88 with placebo; complete-case *P* = 0.053. The sensitivity analysis favored duloxetine (4.06 vs. 4.91; *P* = 0.048).	Breakthrough opioid use was similar between groups.	≥30% pain reduction occurred in 44.1% versus 18.2% (*P* = 0.02), and ≥50% reduction in 32.4% versus 3.0% (*P* = 0.002). Pain-related quality of life also favored duloxetine (*P* = 0.04).	Nausea occurred in 41.2% versus 9.1%, malaise in 20.5% versus 0%, and somnolence in 52.9% versus 45.4%.
Arai et al. (2010) [16]	Median total pain score decreased from 7 to 2 with gabapentin-imipramine, compared with final scores of 4.5, 4, and 5 in comparator groups; *P* = 0.005.	Rescue morphine use was 8 mg/day with combination therapy versus 25-30 mg/day in comparator groups; *P* = 0.008.	Median paroxysmal pain episodes decreased to 1/day with combination therapy versus 3-4/day in comparator groups; *P* < 0.001.	Drowsiness occurred across groups. Three patients receiving higher-dose gabapentin discontinued because of severe dizziness.
Banerjee et al. (2013) [17]	Final VAS scores were 5.56 with gabapentin-tramadol and 5.48 with amitriptyline-tramadol; *P* = 0.482. No significant between-group pain differences were observed.	Rescue analgesia was required by 6 patients receiving gabapentin and 8 receiving amitriptyline.	No significant between-group differences were observed in VRS, percentage pain relief, or global pain scores.	Adverse events occurred in 12 versus 15 patients. Postural hypotension (2 vs. 9; *P* = 0.020) and dry mouth (2 vs. 8; *P* = 0.040) were more frequent with amitriptyline; one patient developed urinary retention.
Chen et al. (2016)^a^ [18]	Pain was controlled in both groups, but detailed between-group VAS estimates and a clearly prespecified primary pain endpoint were not reported.	Mean oxycodone dose was lower with gabapentin at 3 months (33.4 ± 11.0 vs. 58.0 ± 15.2 mg/day; *P* < 0.001) and 6 months (50.7 ± 11.4 vs. 72.7 ± 18.6 mg/day; *P* < 0.001).	Quality-of-life scores favored gabapentin at 3 months (46.8 ± 4.5 vs. 43.5 ± 4.6; *P* = 0.007) and 6 months (46.5 ± 4.8 vs. 41.4 ± 4.3; *P* < 0.001). Treatment costs were also lower at 3 and 6 months.	Nausea/vomiting (10 vs. 18 patients; *P* = 0.038) and constipation (16 vs. 29; *P* < 0.001) were less frequent with gabapentin. Dizziness and drowsiness did not differ significantly.
Sjölund et al. (2013)^b^ [19]	Mean duration-adjusted reduction in worst-pain NRS was -1.53 with pregabalin versus -1.23 with placebo. The trial was stopped early, and only descriptive analyses were performed.	Background opioid therapy remained stable; comparative rescue-opioid outcomes were not clearly reported.	Average pain reduction was -1.24 versus -0.85, and sleep-interference reduction was -1.37 versus -0.63. PGIC improvement occurred in 81.4% versus 70.0%; responder rates numerically favored pregabalin.	Somnolence occurred in 25.0% versus 7.5% and dizziness in 15.3% versus 8.8%. Serious adverse events and deaths were considered unrelated to treatment.
Dou et al. (2017)^c^ [20]	Pain scores remained stable and comparable after morphine titration; the prespecified primary endpoint was morphine-dose reduction rather than pain intensity.	Mean minimum effective morphine dose was 184.4 ± 69.9 mg/day with pregabalin versus 228.7 ± 66.9 mg/day with placebo; *P* < 0.001.	Sleep outcomes improved (*P* < 0.001), and constipation scores were lower (6.4 ± 4.4 vs. 8.6 ± 3.7; *P* = 0.017).	Somnolence (40.0% vs. 10.0%; *P* = 0.004) and dry mouth (20.0% vs. 2.5%; *P* = 0.029) were more frequent with pregabalin.
Garassino et al. (2013) [21]	A ≥33% pain reduction occurred in 76.5% with escalating pregabalin versus 63.9% with escalating oxycodone; OR 1.84, 95% CI 0.65-5.22; *P* = 0.25.	Rescue morphine use was similar between groups (29.4% vs. 28.6%; *P* = 0.59).	Median time to analgesia was 10 versus 11 days. Neuropathic pain and well-being outcomes did not differ significantly.	Constipation, nausea, drowsiness, confusion, and itching were numerically less frequent with pregabalin escalation, but differences were not significant. One patient discontinued because of grade 3 confusion.
Portenoy et al. (2012) [22]	The primary ≥30% pain-response endpoint did not differ significantly between nabiximols and placebo; overall *P* = 0.59.	Scheduled opioid therapy remained stable; no clear opioid-sparing effect was demonstrated.	Continuous responder analysis favored nabiximols overall (*P* = 0.035). Low-dose treatment improved average pain (*P* = 0.006), worst pain (*P* = 0.011), and sleep disruption (*P* = 0.003).	Discontinuation rates were 14.3%, 17.2%, and 27.8% in the low-, medium-, and high-dose groups versus 17.6% with placebo. Common events included nausea, dizziness, somnolence, and vomiting.
Lichtman et al. (2018) [23]	Median improvement in average pain was 10.7% with nabiximols versus 4.5% with placebo; treatment difference 3.41%, 95% CI 0.00-8.16; ITT *P* = 0.0854.	Opioid consumption remained unchanged.	Per-protocol improvement was 15.5% versus 6.3%; *P* = 0.0378. Sleep disruption favored nabiximols (*P* = 0.027), although secondary analyses were unadjusted.	Treatment-related adverse events occurred in 35.2% versus 20.7%. Nausea (8.5% vs. 5.1%) and dizziness (7.5% vs. 2.5%) were most frequent.
Pasutharnchat et al. (2020) [24]	≥30% pain-response rates were similar at 12 hours (65% vs. 70%; *P* = 0.736), 24 hours (80% vs. 75%; *P* > 0.999), 36 hours (85% vs. 80%; *P* > 0.999), and 48 hours (65% vs. 60%; *P* = 0.744).	Median cumulative morphine consumption was 25.5 mg with nefopam versus 37 mg with placebo; *P* = 0.499.	No significant differences were observed in hemodynamic measures or other reported secondary outcomes.	Nausea, vomiting, sweating, dry mouth, dizziness, tachycardia, and drowsiness were uncommon and comparable between groups.

Antidepressant and Gabapentin-Based Strategies

Four trials evaluated antidepressant or gabapentin-based combination strategies [15-18]. Matsuoka et al. evaluated duloxetine added to existing pregabalin and opioid therapy in patients with cancer-related neuropathic pain. At Day 10, the mean average pain score was lower with duloxetine than with placebo, although the primary complete-case comparison did not reach statistical significance (4.03 vs. 4.88; *P* = 0.053). A baseline-observation-carried-forward sensitivity analysis favored duloxetine (4.06 vs. 4.91; *P* = 0.048). Clinically meaningful responder outcomes also favored duloxetine: at least 30% pain reduction occurred in 44.1% of participants receiving duloxetine compared with 18.2% receiving placebo (*P* = 0.02), while at least 50% pain reduction occurred in 32.4% and 3.0%, respectively (*P* = 0.002). Breakthrough opioid use was similar between groups. Nausea and malaise were more frequent with duloxetine [15].

Arai et al. compared low-dose gabapentin plus imipramine with low-dose gabapentin alone, higher-dose gabapentin alone, and imipramine alone. After seven days, the median total pain score decreased from 7 to 2 with combination therapy, compared with final scores of 4.5, 4, and 5 in the respective comparator groups (*P* = 0.005). Median daily paroxysmal pain episodes were also lower with combination treatment (*P* < 0.001). Rescue morphine requirements were 8 mg/day in the combination group compared with 25-30 mg/day in the comparator groups (*P* = 0.008). Three participants receiving higher-dose gabapentin discontinued treatment because of severe dizziness [16].

In contrast, Banerjee et al. found no statistically significant difference between gabapentin plus tramadol and amitriptyline plus tramadol over six months. Final VAS scores were 5.56 and 5.48, respectively (*P* = 0.482), with no significant between-group differences in other measures of pain relief or rescue-analgesic use. Postural hypotension and dry mouth were more frequent with amitriptyline [17].

Chen et al. compared gabapentin plus controlled-release oxycodone with placebo plus controlled-release oxycodone in 70 patients with severe cancer pain, of whom 60 were included in the analysis. Although detailed comparative pain-intensity results were not reported, mean oxycodone requirements were similar between groups at one week and one month but were significantly lower with gabapentin at three months (33.4 ± 11.0 vs. 58.0 ± 15.2 mg/day; *P* < 0.001) and six months (50.7 ± 11.4 vs. 72.7 ± 18.6 mg/day; *P* < 0.001). Quality-of-life scores also favored gabapentin at three months (46.8 ± 4.5 vs. 43.5 ± 4.6; *P* = 0.007) and six months (46.5 ± 4.8 vs. 41.4 ± 4.3; *P* < 0.001). Nausea and vomiting (10 vs. 18 participants; *P* = 0.038) and constipation (16 vs. 29; *P* < 0.001) were less frequent with gabapentin, while dizziness and drowsiness did not differ significantly [18]. 

Pregabalin-Opioid Strategies

Three trials evaluated strategies combining pregabalin with opioid treatment [19-21]. Sjölund et al. randomized 152 patients with cancer-induced bone pain despite optimized stable opioid therapy to flexible-dose pregabalin or placebo. The study was terminated early after an interim analysis, and only descriptive analyses were performed. The mean duration-adjusted reduction in worst-pain NRS favored pregabalin (-1.53 vs. -1.23). Reductions in average pain (-1.24 vs. -0.85) and sleep interference (-1.37 vs. -0.63) also numerically favored pregabalin. At Week 4, 81.4% of pregabalin-treated participants reported improvement on the Patient Global Impression of Change compared with 70.0% receiving placebo, and the proportions achieving at least 30% or 50% pain reduction were numerically greater with pregabalin. Somnolence (25.0% vs. 7.5%) and dizziness (15.3% vs. 8.8%) were more frequent with pregabalin [19].

In the crossover trial by Dou et al., pregabalin reduced the morphine dose required to maintain comparable pain control. The mean minimum effective morphine dose was 184.4 mg/day during pregabalin treatment compared with 228.7 mg/day during placebo treatment (*P *< 0.001). Pregabalin also improved sleep disturbance, sleep quantity, and the overall sleep-problems index (*P* < 0.001). Constipation scores were lower during pregabalin treatment (*P* = 0.017), while somnolence and dry mouth occurred more frequently [20].

Garassino et al. compared fixed-dose oxycodone with escalating pregabalin against fixed-dose pregabalin with escalating oxycodone. A pain reduction of at least 33% was achieved by 76.5% of participants receiving pregabalin escalation and 63.9% receiving oxycodone escalation; however, the difference was not statistically significant (odds ratio (OR) 1.84, 95% confidence interval (CI) 0.65-5.22; *P* = 0.25). Median time to analgesia was 10 and 11 days, respectively. Rescue-morphine use, neuropathic pain scores, and patient well-being did not differ significantly between groups [21].

Nabiximols and Opioid Therapy

Two placebo-controlled trials evaluated nabiximols as an adjunct to established opioid therapy [22,23]. Neither trial met its prespecified primary efficacy endpoint. Portenoy et al. evaluated low-, medium-, and high-dose nabiximols in patients with advanced cancer and chronic pain inadequately controlled by stable opioid treatment. The primary endpoint of at least 30% pain reduction did not differ significantly between nabiximols and placebo (*P* = 0.59). A secondary continuous responder analysis favored nabiximols overall (*P* = 0.035), with effects observed in the low- and medium-dose groups but not in the high-dose group. Low-dose nabiximols improved average pain, worst pain and sleep disruption compared with placebo. Adverse events and treatment discontinuation increased with dose [22].

Lichtman et al. evaluated self-titrated nabiximols alongside optimized opioid therapy. Median improvement in average pain was 10.7% with nabiximols and 4.5% with placebo, but the primary intention-to-treat comparison was not statistically significant (*P* = 0.0854). The per-protocol analysis favored nabiximols (*P* = 0.0378), and sleep disruption improved relative to placebo (*P* = 0.027). These secondary analyses were exploratory because the primary endpoint was not met. Opioid consumption remained unchanged. Treatment-related adverse events occurred in 35.2% of participants receiving nabiximols and 20.7% receiving placebo, with nausea and dizziness reported more frequently with nabiximols [23].

Nefopam and Morphine

One trial evaluated intravenous nefopam as an adjunct to patient-controlled morphine [24]. The proportions of participants achieving at least 30% pain reduction did not differ significantly between nefopam and placebo at 12, 24, 36, or 48 hours. Median cumulative morphine consumption was numerically lower with nefopam than with placebo (25.5 vs. 37 mg), but the difference was not statistically significant (*P* = 0.499). Adverse-event rates were similar between groups [24].

Overall, duloxetine and selected gabapentin- or pregabalin-based regimens showed the most consistent evidence of potential analgesic benefit, with individual studies also reporting reduced opioid requirements while maintaining pain control. In contrast, nabiximols and nefopam did not demonstrate significant benefit for their primary efficacy outcomes.

Secondary outcomes were reported inconsistently across the included trials. Opioid consumption or rescue-analgesic use was evaluated in most studies, although definitions and measurement methods varied. Pregabalin reduced morphine requirements in the crossover trial by Dou et al. [20], while Chen et al. [18] reported lower oxycodone requirements with gabapentin after three and six months. Sleep outcomes favored pregabalin in Dou et al. [20] and numerically favored pregabalin in the prematurely terminated Sjölund trial [19]; selected sleep outcomes also favored nabiximols [23]. Quality-of-life outcomes were infrequently assessed, although Chen et al. [18] reported higher quality-of-life scores with gabapentin at three and six months. Adverse events varied by intervention: duloxetine was associated with more nausea and malaise [15]; amitriptyline with greater anticholinergic and orthostatic effects [17]; pregabalin with somnolence, dizziness, and dry mouth [19,20]; and nabiximols with nausea and dizziness [22,23]. In a study by Chen et al., lower oxycodone exposure with gabapentin was accompanied by lower rates of nausea, vomiting, and constipation [18].

Discussion

This systematic review aimed to evaluate the efficacy and safety of non-opioid or adjuvant analgesics used alongside opioid therapy in adults with chronic cancer pain. In addition to pain intensity and clinically meaningful pain response, the review examined whether these combinations reduced opioid or rescue-analgesic requirements and improved sleep, physical function, quality of life, tolerability, or adverse effects. Ten randomized controlled trials were identified. Selected antidepressant and gabapentinoid-based strategies showed signals of potential benefit in individual trials, particularly in neuropathic cancer pain, through improved pain response or reduced opioid requirements. However, confidence in these findings is limited by inconsistent results, substantial clinical heterogeneity, high risk of bias in several trials, and the evaluation of most interventions in only one relatively small study. The available evidence therefore does not support a single standard multimodal pharmacological regimen.

These findings broadly align with previous reviews of adjuvant and non-opioid analgesics for cancer pain. Vardy and Agar concluded that antidepressants and anticonvulsants may have a role in neuropathic cancer pain, but highlighted methodological limitations, small samples, short follow-up, and uncertainty regarding the longer-term efficacy and safety of non-opioid agents used with strong opioids. They similarly called for larger and longer-duration studies [23]. Previous systematic reviews of adjuvant analgesics and non-opioid drug combinations also found limited and heterogeneous evidence supporting individual agents or specific combinations [11,12,23]. In contrast to broader reviews that included non-opioid monotherapy, acute pain, or combinations without opioids, the present review focused specifically on randomized trials in which a non-opioid or adjuvant analgesic was administered alongside opioid therapy for ongoing cancer-related pain requiring regular analgesic therapy. It also considered opioid consumption and patient-centered outcomes alongside pain intensity. Nevertheless, the present findings reinforce rather than substantially resolve the uncertainty identified by earlier reviews: potentially beneficial signals exist, particularly for antidepressants and gabapentinoids, but the evidence remains too fragmented to establish comparative effectiveness or recommend a standard combination [11,12,25].

This continuing uncertainty is clinically important because cancer pain remains common despite advances in oncology and supportive care. A recent meta-analysis found that 44.5% of patients across the cancer trajectory experienced pain and 30.6% experienced moderate-to-severe pain; prevalence remained particularly high among patients with advanced, metastatic, or terminal disease [26]. During and shortly after curative cancer treatment, pooled pain prevalence has been estimated at approximately 40%, with marked heterogeneity related partly to differences in pain measurement [27]. Cancer pain may also persist into survivorship and adversely affect physical function, emotional well-being, sleep, treatment adherence, and quality of life [28].

Effective management is complicated by heterogeneous pain mechanisms, including nociceptive, neuropathic, visceral, bone, treatment-related, and mixed pain, which may coexist within the same patient. Opioids remain central to moderate-to-severe cancer pain management, but dose escalation may be restricted by constipation, nausea, sedation, cognitive effects, and other toxicities. Adjuvant treatment may therefore improve analgesia or reduce opioid exposure, but can also introduce additional adverse effects, drug interactions, monitoring requirements, and treatment burden. Wider barriers include inconsistent pain assessment, limited clinician knowledge, concerns about opioid adverse effects and dependence, patients’ reluctance to report pain, and disparities in access to specialist supportive and palliative care [28]. Contemporary evidence suggests that undertreatment remains substantial, with one updated review reporting a weighted prevalence of approximately 40% [29]. These challenges support a mechanism-based approach but also emphasize the need for stronger evidence to guide the selection and sequencing of adjunctive pharmacotherapy.

Duloxetine showed a potential signal of benefit in one small trial. Although the primary comparison of mean pain scores did not reach statistical significance, clinically meaningful responder rates favored duloxetine. These findings suggest a possible role in refractory neuropathic cancer pain, but the small sample, 10-day treatment period, and higher incidence of nausea and malaise limit conclusions regarding longer-term effectiveness and tolerability [15].

Gabapentin- and imipramine-based combinations also showed possible benefit. Low-dose gabapentin plus imipramine improved short-term pain outcomes and reduced rescue-morphine use, but the trial was small, open-label and limited to seven days [16]. In contrast, gabapentin-tramadol and amitriptyline-tramadol provided comparable analgesia over six months, with tolerability favoring gabapentin because of fewer anticholinergic and orthostatic effects [17]. Chen et al. additionally reported lower long-term oxycodone requirements and improved quality-of-life scores with gabapentin [18]. However, interpretation is limited by incomplete comparative pain reporting, uncertainty regarding the analysis population, and the absence of a clearly reported prespecified primary pain endpoint [18]. Taken together, these studies support the potential utility of gabapentin-based treatment but do not establish its superiority over other adjuvant strategies.

Pregabalin-based strategies demonstrated possible analgesic and opioid-sparing effects. In the crossover trial by Dou et al., pregabalin reduced the morphine dose required to maintain pain control and improved sleep. Lower constipation scores may have reflected reduced morphine exposure rather than a direct effect of pregabalin, while somnolence and dry mouth were more frequent [20]. Sjölund et al. reported numerical improvements in pain and sleep interference in cancer-induced bone pain, but the study was terminated early, and formal comparative analyses were not completed [19]. Garassino et al. similarly found a numerically greater pain response when pregabalin was escalated rather than oxycodone, although the difference was not statistically significant and the confidence interval was wide [21]. Collectively, these findings suggest that optimizing an adjuvant while limiting opioid escalation may be feasible in selected patients, but superiority over conventional opioid titration has not been established.

Evidence for nabiximols and nefopam was less convincing. Neither nabiximols trial met its prespecified primary efficacy endpoint [22,23]. Although some continuous-responder, per-protocol, sleep, and lower-dose findings favored nabiximols, these analyses were secondary or exploratory and should not override the negative primary outcomes. Nabiximols was also associated with nausea, dizziness, and increasing discontinuation at higher doses [22,23]. Nefopam did not significantly improve pain-response rates or reduce morphine consumption [24]. Its small sample and 48-hour assessment period limit conclusions, but the available evidence does not currently support routine use as an opioid adjunct for chronic cancer pain [24].

This review has several strengths. It was prospectively registered with PROSPERO, followed PRISMA 2020, searched three major databases, and used independent study selection, data extraction, and RoB 2 assessment by two reviewers. By restricting inclusion to randomized controlled trials evaluating non-opioid or adjuvant analgesics alongside opioids, the review addressed a clinically focused question distinct from reviews of non-opioid monotherapy or broader cancer-pain interventions. Opioid consumption, rescue-medication use, sleep, quality of life, tolerability, and adverse events were considered alongside pain outcomes.

Several limitations should be acknowledged. Only English-language published studies were included, and gray literature was not searched; therefore, language and publication bias cannot be excluded. In addition, requiring opioid-related terminology may have reduced search sensitivity where background opioid therapy was not clearly reported in the title, abstract, or indexing. The search strategy was not formally validated against a prespecified set of sentinel studies. Meta-analysis and formal assessment of publication bias were not feasible because of substantial clinical and methodological heterogeneity and the small number of comparable studies.

A formal certainty-of-evidence assessment was not performed. Confidence in many of the findings is nevertheless limited by generally small sample sizes, short treatment periods, variable outcome definitions and assessment time points, and high risk of bias in seven of the 10 trials. Considerable heterogeneity was also present in cancer populations, pain mechanisms, baseline opioid regimens, pharmacological interventions, comparator treatments, treatment duration, and outcome measurement. In addition, an operational definition of established chronic cancer pain was required because several trials did not explicitly report pain duration, which may have introduced further clinical heterogeneity. The observed benefits should therefore be interpreted cautiously and as preliminary signals rather than definitive evidence of treatment effect.

Overall, the evidence base remained concentrated in neuropathic pain, with relatively little evaluation of nociceptive, visceral, musculoskeletal, mixed, or persistent treatment-related pain. Outcomes beyond pain intensity were also assessed inconsistently. Although some trials reported opioid consumption, sleep, and quality of life, functional outcomes and longer-term patient-centered measures were uncommon. Consequently, the applicability of these findings to the broader population of people living with and beyond cancer remains limited.

Based on the available evidence, clinicians should adopt an individualized, mechanism-based approach rather than routinely prescribing a specific multimodal pharmacological regimen for chronic cancer pain. The most consistent signals of potential benefit were seen in studies involving neuropathic cancer pain. Selected antidepressants or gabapentinoids may be considered on an individual basis in patients with neuropathic cancer pain when opioid therapy provides inadequate relief or further escalation is limited by toxicity. Treatment decisions should account for pain mechanism, renal and hepatic function, comorbidities, concomitant medications, previous treatment response, functional goals, and adverse-effect profiles. When these agents are used, they should be introduced and titrated cautiously, with monitoring for class-specific adverse effects, including nausea, anticholinergic or orthostatic effects, somnolence, and dizziness. Current evidence remains insufficient to recommend routine use of nabiximols or nefopam as opioid adjuncts.

Future trials should be adequately powered, use longer treatment and follow-up periods, and apply standardized definitions of clinically meaningful pain response. Consistent reporting of opioid consumption, rescue-medication use, adverse events, physical function, sleep, and quality of life would improve comparability. Further studies are particularly needed in nociceptive, visceral, musculoskeletal, mixed, bone, and persistent treatment-related pain, including among cancer survivors.

## Conclusions

Across 10 randomized controlled trials, selected antidepressant and gabapentinoid-based strategies showed potential benefit when added to opioids, particularly for neuropathic cancer pain. One duloxetine trial reported favorable clinically meaningful pain-response rates, although it was not statistically significant. Individual gabapentin- and pregabalin-based studies also demonstrated possible analgesic or opioid-sparing effects. Evidence for nabiximols and nefopam was inconclusive. Given the heterogeneity of the included studies and the high risk of bias in several trials, the available evidence is insufficient to identify a standard multimodal pharmacological regimen. Treatment should therefore remain individualized according to pain mechanism, previous response, comorbidities, and tolerability.

## Appendices

Appendix A 

**Table 3:** EMBASE search strategy Supplementary Table S1. EMBASE search strategy

Line	Search strategy	Results (n)
1	(Cancer* or Tumo?r* or Neoplas* or oncolog* or metasta* or maligna* or gliom* or sarcom* or chemotherap* or melanom* or leukaem* or leukem* or lymphom* or mesotheliom* or radiotherap* or immunotherap* or brachytherap*).ab,ti.	7,098,582
2	malignant neoplasm/	272,636
3	1 or 2	7,141,999
4	("Combination Therap*" or Gabapentinoid* or NSAIDS or "non-steroidal anti-inflammatory drug*" or "adjunct therap*" or "Anti depressants" or "Anti convulsant" or "Steroid*" or "corticosteroid*").ab,ti.	803,850
5	gabapentin/ or pregabalin/ or analgesic agent/ or nonsteroid antiinflammatory agent/ or antidepressant agent/ or fluoxetine/ or amitriptyline/ or serotonin uptake inhibitor/ or phenytoin/ or valproic acid/ or anticonvulsive agent/ or steroid/ or corticosteroid/	1,224,779
6	4 or 5	1,664,049
7	("Single Therap*" or Opioid* or Monotherap*).ab,ti.	365,907
8	monotherapy/ or opiate/	341,535
9	7 or 8	495,419
10	("Chronic Pain" or "Acute Pain" or (pain adj3 Reduc*) or "Neuropathic Pain").ab,ti.	233,502
11	neuropathic pain/ or chronic pain/ or neuropathy/	208,860
12	10 or 11	337,935
13	3 and 6 and 9 and 12	3,739
14	limit 13 to (english language and yr="2010 -Current" and (adult <18 to 64 years> or aged <65+ years>))	1,456
15	limit 13 to (english language and randomized controlled trial and yr="2010 -Current" and (adult <18 to 64 years> or aged <65+ years>))	277
16	("Terminal Care" or "End of Life Care").ab,ti.	95,775
17	15 not 16	259
18	limit 17 to dd=19000101-20250831	92

Appendix B 

**Table 4:** MEDLINE (Ovid) search strategy Supplementary Table S2. MEDLINE (Ovid) search strategy The strategy contains terms for both the EMBASE and MEDLINE databases. Lines 1-18 show the strategy as it was run in EMBASE. The following lines show the process of adapting the strategy between databases searched on Ovid platform. From line 19 onward, the strategy was correctly adapted for MEDLINE by replacing the Embase-specific Emtree terms with the appropriate MEDLINE MeSH and recombining these with relevant free-text terms. This final strategy yielded 36 records and was the strategy used for study selection and reflected in the PRISMA diagram

Line	Search strategy	Results (n)
1	(Cancer* or Tumo?r* or Neoplas* or oncolog* or metasta* or maligna* or gliom* or sarcom* or chemotherap* or melanom* or leukaem* or leukem* or lymphom* or mesotheliom* or radiotherap* or immunotherap* or brachytherap*).ab,ti.	5,116,330
2	malignant neoplasm/	572,201
3	1 or 2	5,200,106
4	("Combination Therap*" or Gabapentinoid* or NSAIDS or "non-steroidal anti-inflammatory drug*" or "adjunct therap*" or "Anti depressants" or "Anti convulsant" or "Steroid*" or "corticosteroid*").ab,ti.	519,596
5	gabapentin/ or pregabalin/ or analgesic agent/ or nonsteroid antiinflammatory agent/ or antidepressant agent/ or fluoxetine/ or amitriptyline/ or serotonin uptake inhibitor/ or phenytoin/ or valproic acid/ or anticonvulsive agent/ or steroid/ or corticosteroid/	285,813
6	4 or 5	726,487
7	("Single Therap*" or Opioid* or Monotherap*).ab,ti.	223,486
8	monotherapy/ or opiate/	443
9	7 or 8	223,793
10	("Chronic Pain" or "Acute Pain" or (pain adj3 Reduc*) or "Neuropathic Pain").ab,ti.	150,673
11	neuropathic pain/ or chronic pain/ or neuropathy/	49,482
12	10 or 11	166,621
13	3 and 6 and 9 and 12	701
14	limit 13 to (english language and yr="2010 -Current" and (adult <18 to 64 years> or aged <65+ years>))	489
15	limit 13 to (english language and randomized controlled trial and yr="2010 -Current" and (adult <18 to 64 years> or aged <65+ years>))	43
16	("Terminal Care" or "End of Life Care").ab,ti.	58,318
17	15 not 16	42
18	limit 17 to dd=19000101-20250131	42
19	exp Neoplasms/	4,290,882
20	Gabapentin/ or Pregabalin/ or Analgesics/ or Anti-Inflammatory Agents, Non-Steroidal/ or Antidepressive Agents/ or Fluoxetine/ or Amitriptyline/ or Receptors, Serotonin/ or Selective Serotonin Reuptake Inhibitors/ or Serotonin/ or Serotonin Agents/ or "Serotonin and Noradrenaline Reuptake Inhibitors"/ or Phenytoin/ or Valproic Acid/ or Anticonvulsants/ or Steroids/	406,973
21	Opiate Alkaloids/	443
22	Chronic Pain/ or Neuralgia/	49,482
23	1 or 19	6,080,802
24	4 or 20	865,441
25	7 or 21	223,793
26	10 or 22	166,621
27	23 and 24 and 25 and 26	750
28	limit 27 to (english language and yr="2010 -Current" and "all adult (19 plus years)" and randomized controlled trial)	40
29	28 not 16	39
30	limit 29 to dt=19000101-20250831	36

Appendix C 

**Table 5:** CENTRAL via Cochrane Library search strategy Supplementary Table S3. CENTRAL via Cochrane Library search strategy

Line	Search Strategy	Results (n)
#1	(Cancer* or Tumo?r* or Neoplas* or oncolog* or metasta* or maligna* or gliom* or sarcom* or chemotherap* or melanom* or leukaem* or leukem* or lymphom* or mesotheliom* or radiotherap* or immunotherap* or brachytherap*)	340,634
#2	MeSH descriptor: [Neoplasms] explode all trees	127,280
#3	#1 OR #2	346,520
#4	(Combination NEXT Therap* or Gabapentinoid* or NSAIDS or non-steroidal NEXT anti-inflammatory* or adjunct NEXT therap* or "Anti depressant" or "Anti convulsant" or Steroid* or corticosteroid* or cannabino* or bisphosphonate* or alpha NEXT blocker* or capsaicin or qutenza)	88,907
#5	MeSH descriptor: [Gabapentin] this term only	1,078
#6	MeSH descriptor: [Pregabalin] this term only	1,068
#7	MeSH descriptor: [Analgesics] this term only	6,629
#8	MeSH descriptor: [Steroids] this term only	1,135
#9	MeSH descriptor: [Cannabinoids] explode all trees	1,577
#10	MeSH descriptor: [Adrenergic alpha-Antagonists] this term only	1,157
#11	MeSH descriptor: [Capsaicin] this term only	796
#12	#4 or #5 or #6 or #7 or #8 or #9 or #10 or #11	97,892
#13	(Single NEXT Therap* or Opioid* or Monotherap*)	69,290
#14	MeSH descriptor: [Opiate Alkaloids] this term only	32
#15	#13 or #14	69,301
#16	("Chronic Pain" or "Acute Pain" or (pain NEAR3 Reduc*) or "Neuropathic Pain")	745,581
#17	MeSH descriptor: [Neuralgia] this term only	1,769
#18	MeSH descriptor: [Chronic Pain] this term only	4,809
#19	#16 or #17 or #18	745,684
#20	#3 and #12 and #15 and #19 with publication date between Jan 2010 and Aug 2025, in CENTRAL	394

Appendix D 

**Table 6:** Detailed Risk of Bias 2 assessments of included studies Supplementary table S4. Domain-level judgements were made using the Cochrane RoB 2 tool. D1: bias arising from the randomisation process; D2: bias due to deviations from intended interventions; D3: bias due to missing outcome data; D4: bias in measurement of the outcome; D5: bias in selection of the reported result. Judgements were classified as low risk of bias, some concerns, or high risk of bias.

Study	Outcome assessed	D1 Randomisation	Crossover: period/carryover	D2 Deviations	D3 Missing data	D4 Outcome measurement	D5 Selective reporting	Overall	Key rationale
Arai et al., 2010	Total pain score at 7 days	Some concerns	N/A	Some concerns	Some concerns	High risk	Some concerns	High risk	Open-label design with subjective pain assessment; allocation concealment and prespecified reporting were not sufficiently clear.
Portenoy et al., 2012	≥30% pain-response endpoint	Low risk	N/A	Low risk	High risk	Low risk	Low risk	High risk	Substantial attrition (360 randomised; 263 completed).
Banerjee et al., 2013	VAS pain outcome	Low risk	N/A	Low risk	High risk	Low risk	Some concerns	High risk	88 participants were randomised and 76 completed six months, with high risk assigned to missing outcome data. The trial was open-label and used patient-reported pain outcomes.
Garassino et al., 2013	≥33% pain reduction at 14 days	Low risk	N/A	Some concerns	Some concerns	High risk	Some concerns	High risk	Computer-generated randomisation was reported. The dose-escalation strategies were not described as blinded and pain was assessed with subjective NRS/diary measures, supporting the high D4 judgement. Attrition was limited but present.
Sjölund et al., 2013	Duration-adjusted reduction in worst-pain NRS	Some concerns	N/A	Low risk	High risk	Low risk	Low risk	High risk	Trial recruitment was terminated early and the planned formal comparative analyses were not completed; outcomes were reported descriptively.
Chen et al., 2016	Cancer pain/VAS outcome (primary pain endpoint unclear)	Some concerns	N/A	Some concerns	Some concerns	Low risk	High risk	High risk	Seventy participants entered the study and 60 were analysed; six were lost to follow-up and four withdrew. VAS was recorded, but detailed comparative VAS results and a clearly specified primary pain endpoint were not reported.
Dou et al., 2017	Minimum effective morphine dose while maintaining pain control	Some concerns	Some concerns	Low risk	Low risk	Low risk	Some concerns	Some concerns	Double-blind randomised placebo-controlled crossover design with 40 randomised, 38 completing, and all 40 included in ITT. Some concerns remain around randomisation reporting, result-selection reporting, and the crossover-specific period.
Lichtman et al., 2018	Improvement in average pain	Some concerns	N/A	Low risk	High risk	Low risk	Low risk	High risk	Of 397 randomised participants, 291 completed; substantial withdrawal/missing efficacy data drives the overall judgement.
Matsuoka et al., 2019	Average pain score at Day 10	Low risk	N/A	Low risk	Low risk	Low risk	Low risk	Low risk	Multicenter double-blind placebo-controlled trial with prespecified analysis, low missing data and sensitivity analysis.
Pasutharnchat et al., 2020	≥30% pain reduction over 48 hours	Low risk	N/A	Low risk	Low risk	Low risk	Low risk	Low risk	Double-blind randomised placebo-controlled trial, prospectively registered, with 40 participants randomised (20 per group) and the published flow/results appearing to include all 40.
